# Spatiotemporal distribution patterns of immature Australasian white sharks (*Carcharodon carcharias*)

**DOI:** 10.1038/s41598-020-66876-z

**Published:** 2020-06-23

**Authors:** Julia L. Y. Spaet, Toby A. Patterson, Russell W. Bradford, Paul A. Butcher

**Affiliations:** 1 Evolutionary Ecology Group, Department of Zoology, University of Cambridge, Downing Street, Cambridge, CB2 3EJ UK; 2 Southern Cross University, Coffs Harbour, New South Wales, 2450 Australia; 3 CSIRO Oceans and Atmosphere, Hobart, TAS 7004 Australia; 4 NSW Fisheries, NSW Department of Primary Industries, National Marine Science Centre, Coffs Harbour, New South Wales, 2450 Australia

**Keywords:** Animal migration, Behavioural ecology, Conservation biology

## Abstract

In Australian and New Zealand waters, current knowledge on white shark (*Carcharodon carcharias*) movement ecology is based on individual tracking studies using relatively small numbers of tags. These studies describe a species that occupies highly variable and complex habitats. However, uncertainty remains as to whether the proposed movement patterns are representative of the wider population. Here, we tagged 103 immature Australasian white sharks (147–350 cm fork length) with both acoustic and satellite transmitters to expand our current knowledge of population linkages, spatiotemporal dynamics and coastal habitats. Eighty-three sharks provided useable data. Based on individual tracking periods of up to 5 years and a total of 2,865 days of tracking data, we were able to characterise complex movement patterns over ~45° of latitude and ~72° of longitude and distinguish regular/recurrent patterns from occasional/exceptional migration events. Shark movements ranged from Papua New Guinea to sub-Antarctic waters and to Western Australia, highlighting connectivity across their entire Australasian range. Results over the 12-year study period yielded a comprehensive characterisation of the movement ecology of immature Australasian white sharks across multiple spatial scales and substantially expanded the body of knowledge available for population assessment and management.

## Introduction

Defining the extent of movements and the degree of population connectivity is an important prerequisite for an improved ecological understanding of a species and hence for designing effective conservation measures^1^. For wide ranging species, long-term monitoring of a representative proportion of a population^2–6^ is crucial for interpreting whether the apparent measures of abundance or rates of encounter are related to abundance changes or shifts in spatial distribution^7,8^. For marine species, large scale satellite and acoustic telemetry studies have provided many insights into the spatial resolution of movements and long-distance migrations of predators, often challenging our existing understanding of a species’ spatial distribution and habitat use^9,10^. White sharks (*Carcharodon carcharias*, Linnaeus, 1758) are an exemplar species in this sense. Previously considered to mainly inhabit coastal shelf waters in cold and temperate ocean regions^11^, studies using electronic telemetry have shown that individuals undertake extensive oceanic and continental-scale migrations, spending considerable time in the open ocean and in subtropical and tropical habitats^5,6,12–14^. These observations have been important in determining the degree of connectivity and the scale of white shark spatial usage.

White sharks are globally listed as Vulnerable based on ICUN Red List criteria^15^ and have been afforded protection under various national jurisdictions and international treaties, such as the Convention on International Trade in Endangered Species of Wild Fauna and Flora (CITES) Appendix II listing and the Convention on the Conservation of Migratory Species of Wild Animals (CMS). This has fostered wide-ranging research and conservation efforts over much of their global distribution^16^. Under Australia’s Environmental Protection of Biodiversity and Conservation (EPBC) Act the species is listed as threatened and conservation objectives at a national level are formulated under the species recovery plan^17^. A key priority of research under this plan is to improve our knowledge of important habitat within Australian waters and the degree of connectivity between Australia and surrounding regions.

**Figure 1:**
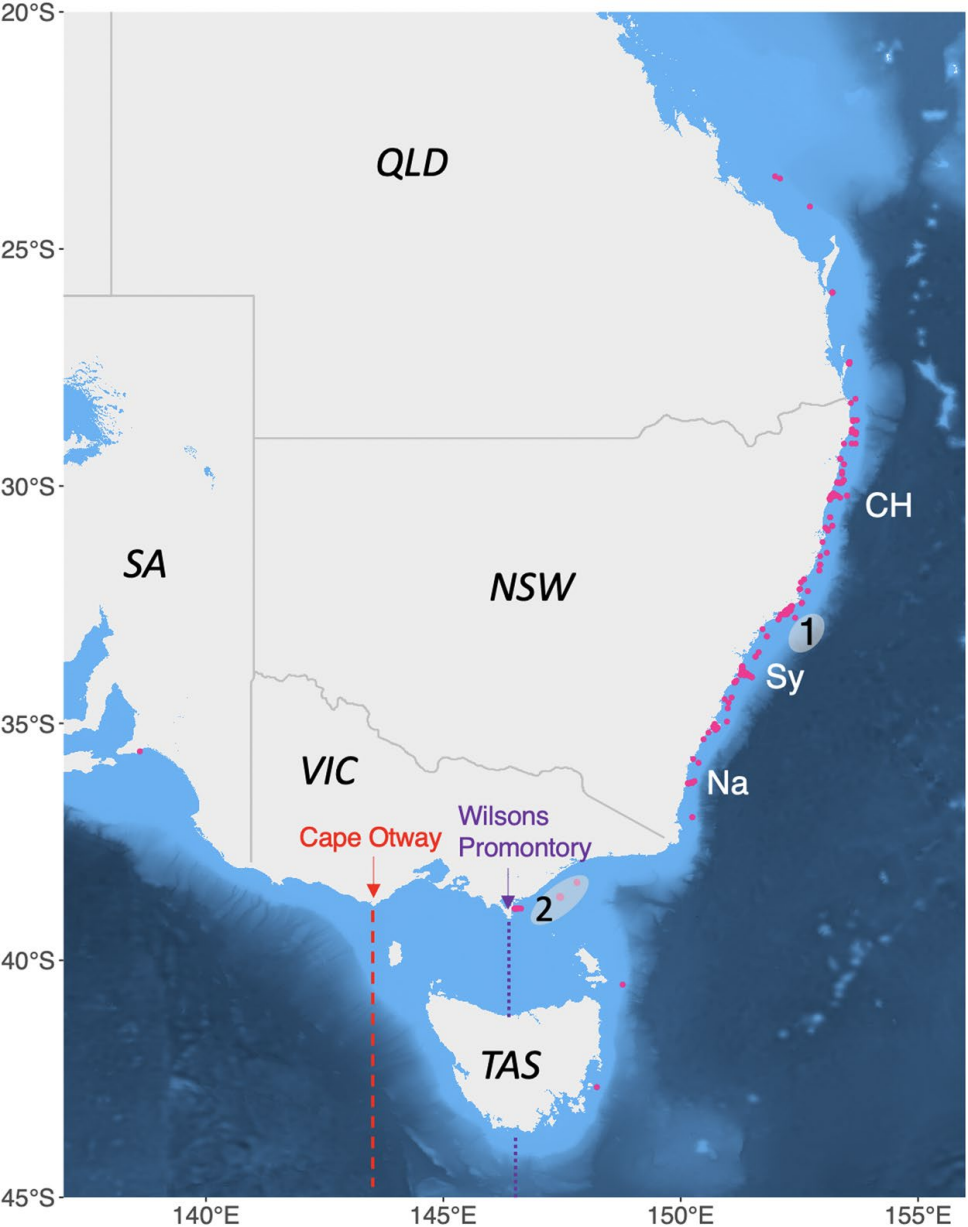
Distribution of acoustic receiver arrays. Pink dots indicate locations of individual receivers off the east/south-east coast of Australia  that provided data for the present study. Integrated Marine Observing System (IMOS) cross shelf lines of receivers are positioned at Coffs Harbour (CH), Sydney (Sy) and Narooma (Na). Grey shaded areas indicate nursery areas previously identified along the eastern/south-eastern Australian coast – 1: Port Stephens nursery area; 2: Corner Inlet nursery area. The dotted purple line indicates the previously hypothesized line of separation between the eastern Australasian and southern-western Australasian sub-populations. The dashed red line indicates the western limit of typical shark movements observed in this study. Map was generated using the marmap package in R^67^.

White sharks in waters surrounding eastern Australia and New Zealand (hereafter referred to as eastern Australasian white sharks) comprise a single population estimated to include ca. 2,500–6,750 individuals^18^. Within the eastern Australasian white shark population distribution, two juvenile nurseries have been identified: one centred around Port Stephens in New South Wales; the other in the Corner Inlet/Ninety-mile Beach region of Victoria^19^ (Fig. 1). Temporary residency of these areas has been hypothesized to be linked with seasonal upwelling and the associated aggregation of various teleost species, suggesting foraging and regional environmental conditions as potential drivers for seasonal residency^19^. Previous acoustic and satellite tracking studies off eastern Australia have revealed a range of movements and possible seasonal patterns within this population^20–23^. While movements of juvenile eastern Australasian white sharks observed in previous studies were mostly confined to coastal waters^19,20,24^, a number of juveniles and adults of both sexes were shown to undertake long-distance migrations from subtropical and tropical locations to temperate^22,23,25^ and sub-Antarctic areas^21^. Based on these previous observations, three north-south migratory corridors have been proposed: (1) from southwest South Island, New Zealand; to southern Queensland; (2) from the Chatham Islands, New Zealand; along northeast North Island, New Zealand; to New Caledonia^21^; and (3) along the east coast of Australia^20^ (Supplementary Figure S1). The identification of frequently used corridors is essential to implement effective fisheries management policies for wide-ranging species that require international conservation efforts. However, uncertainty remains as to whether the described corridors are widely used by Australasian white sharks.

Here, we report on a long-term, multi-tagging study of immature eastern Australasian white sharks. The study was conducted as part of a New South Wales Government funded extensive tagging programme aimed at filling important knowledge gaps in our understanding of regional white shark spatial dynamics. During the course of this initiative and a previous sampling effort led by the Commonwealth Scientific and Industrial Research Organisation (CSIRO), we collected one of the most extensive combined acoustic and satellite tagging datasets on white shark movements. This paper provides a first description of this dataset; further comprehensive analyses to investigate drivers of the described patterns are intended for future publications. Here, we investigate the timing and extent of movement patterns of immature eastern Australasian white sharks, compare migration and occurrence patterns to those described in previous studies, and discuss potential factors underlying inter-individual and sex-based differences in movement patterns.

## Results

Between 08 October 2007 and 18 July 2019, a total of 103 immature white sharks were dual-tagged with acoustic transmitters (Vemco V16-6L, Vemco, Bedford, Nova Scotia, Canada) and satellite-linked radio transmitting (SLRT; Wildlife Computers SPOT tags) tags. Two additional sharks were tagged with SLRT tags only. Out of those, 102 sharks were tagged in New South Wales coastal shelf waters and one was tagged in Corner Inlet, Victoria (Supplementary Table S1). Sharks ranged in fork length (FL) from 147 to 350 cm (mean 224.3 cm, SD 39.0). A total of 87 sharks were included in the final analyses for this project. Those which were excluded included three, which due to unknown reasons, failed to be detected on any acoustic receiver or ARGOS satellite, and a further 13 with <20 detections, which resulted in poor resolution of the Kalman filtering model (see methods). Of the 87 remaining sharks, 94% (n = 82) were juveniles (155–280 cm FL; 36 male, 46 female), four were sub-adults (281–350 cm FL; all male) and one was a young-of-the-year (YOY; 147 cm FL; male) at the time of tagging based on the life history definitions provided by Bruce and Bradford^19^. The sex ratio was skewed towards females 50:37. Mean time at liberty (number of days between tagging and last detection) was 540 d (range: 38–1816 d, median: 428) and 55 sharks were tracked for durations >1 year (Supplementary Table S1).

### Spatial scale of movements

**Figure 2:**
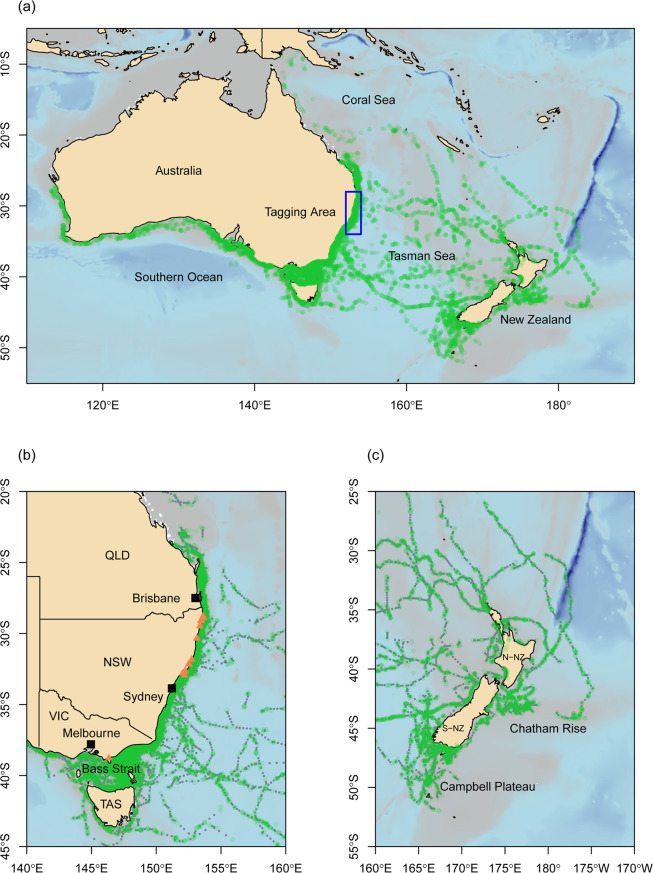
Spatial scale of movements. Smoothed and interpolated telemetry tracks of 87 immature white sharks tagged off eastern Australia from a combination of satellite-linked radio, acoustic and recapture records, collected between 08 October 2007 – 30 September 2019. Green dots represent retained Argos positions and acoustic detections, blue dots represent Kalman filter/smoother interpolation estimates on a 6 hour time step. (**a**) Broad-scale range of movements (shown are retained Argos and acoustic detections only) (**b**) Regional movements along the Australian east coast and into the Bass Strait. Orange triangles indicate tagging locations. (**c**) Regional movements in waters surrounding New Zealand. Maps were generated using the marmap package in R^67^.

Movements of immature white sharks were estimated over a 12-year period between 2007 and 2019. Movements ranged from southern Papua-New Guinea to Western Australia and across the Tasman Sea to sub-Antarctic waters southeast of New Zealand, spanning ~45 degrees of latitude and ~72 degrees of longitude (Fig. 2a–c).

### Seasonal and spatial distribution patterns

**Figure 3:**
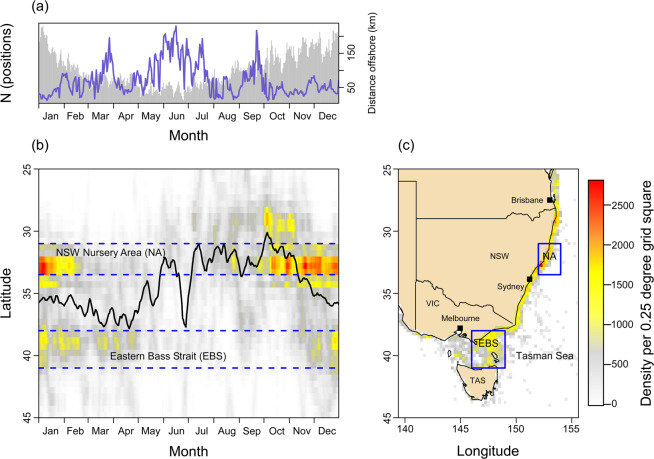
Seasonal migration cycle and residency patterns. (**a**) Number of Daily Average Positions (DAPs, grey bars) and average distance offshore (blue line) by month. (**b**) DAPs combined across all individuals and all years by latitude and month. The weighted average latitude is shown by the black line. The coloured points grading from grey (low density) to red (high density) display the seasonal change in habitat utilization intensity of immature eastern Australasian white sharks. (**c**) DAPs combined across all individuals and all years within eastern and south-eastern Australian waters and the eastern Bass Strait area. Map was generated using the marmap package in R^67^.

The number of Daily Average Positions (DAPs) revealed indications of a seasonal presence/absence pattern in eastern Australasian coastal habitats with peak abundances in the austral spring and summer (October – February) that gradually decreased to a trough in shark presence from March – June before they increased again over the austral winter (July – August) (Fig. 3a). A corresponding onshore-offshore movement pattern was indicated by the average distance offshore relative to time of year, with distances offshore being shortest from October to January and longest from May to July (Fig. 3a).

**Figure 4:**
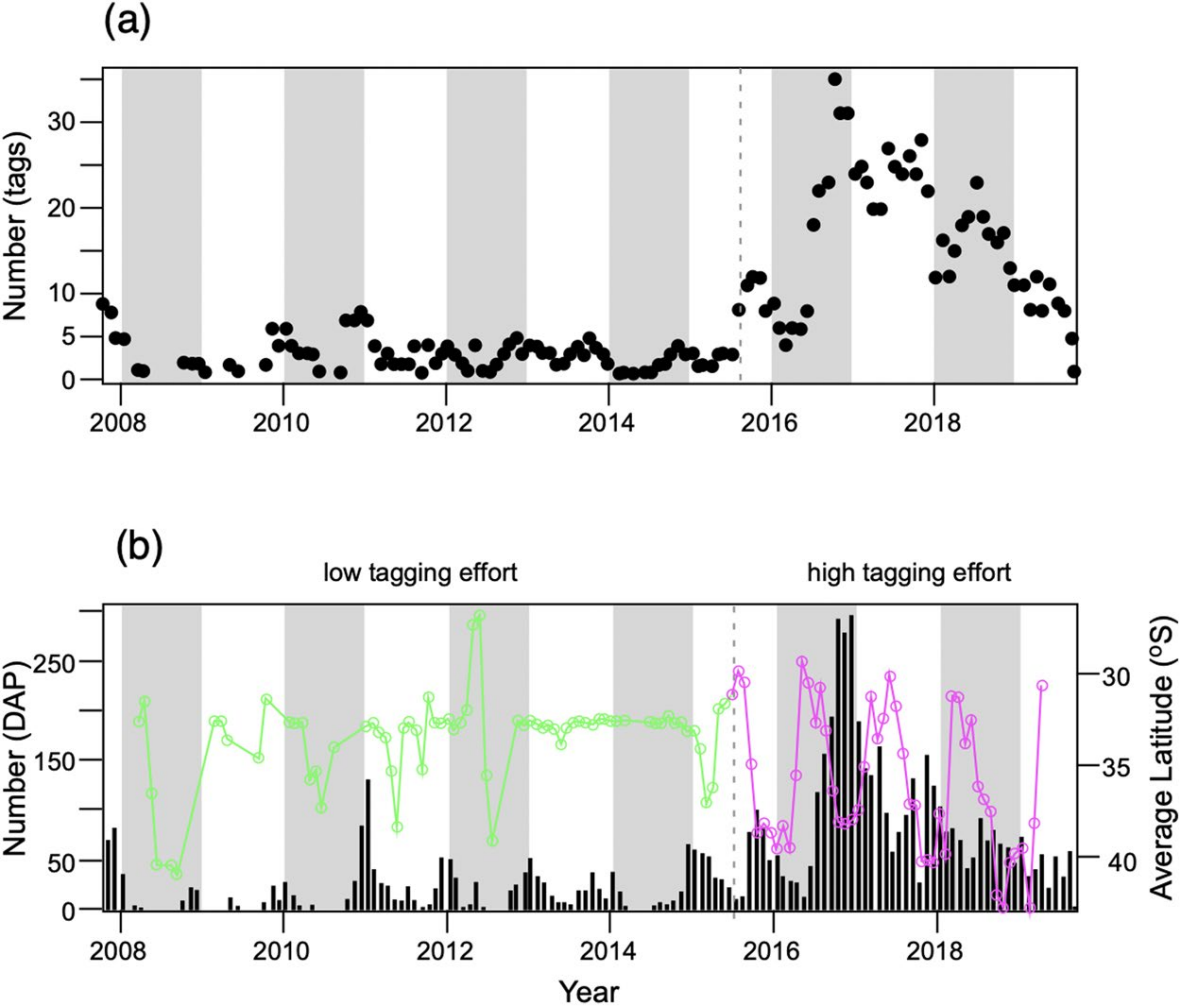
Tagging effort and detection of seasonal migration cycle. (**a**) Total number of tags detected by year and month. (**b**)Total number of satellite and acoustic DAPs by year and month (vertical bars) with the average latitudinal position superimposed (green and pink line). The average latitudinal position for the period of initial tagging is given by the green line. The dashed vertical line shows the initiation of a large-scale tagging programme by the NSW Government in 2015. The magenta line shows the clear seasonal pattern in average latitude from a larger tag deployment. The grey shading indicates years.

The total latitudinal range used by immature white sharks was large (45 degrees). Yet, 50% (n = 43) of all tracked individuals maintained a restricted (ca. 1000 km) range within ~28°S and ~38°S, corresponding to the area between the Queensland/New South Wales (28.01**°**S, 153.39°E) and the New South Wales/Victoria (37.52°S, 150.02°E) borders. Another 35% (n = 30) extended this range to ~45°S, into waters within Bass Strait and around Tasmania. Across the entire study area, main areas of activity were in New South Wales waters between 30°S and 35°S, followed by the eastern Bass Strait area between 38°S and 41°S (Fig. 3b,c). Within New South Wales waters, the high use zone was primarily occupied from late August to late February. In Victorian waters, it was occupied primarily from December through April, showing some overlap with New South Wales. A seasonal north-south migration pattern along the Australian east coast (Fig. 3b) became particularly evident after mid-2015, as sample size increased from 25 to a total of 87 tags (Fig. 4a,b). Starting in September, tracked sharks spent an increased amount of time in southern Queensland and northern New South Wales (27.5°S) waters, followed by a tendency to gradually move south reaching latitudes between 37.5°S and 42.5°S by March (Fig. 3b).

**Figure 5:**
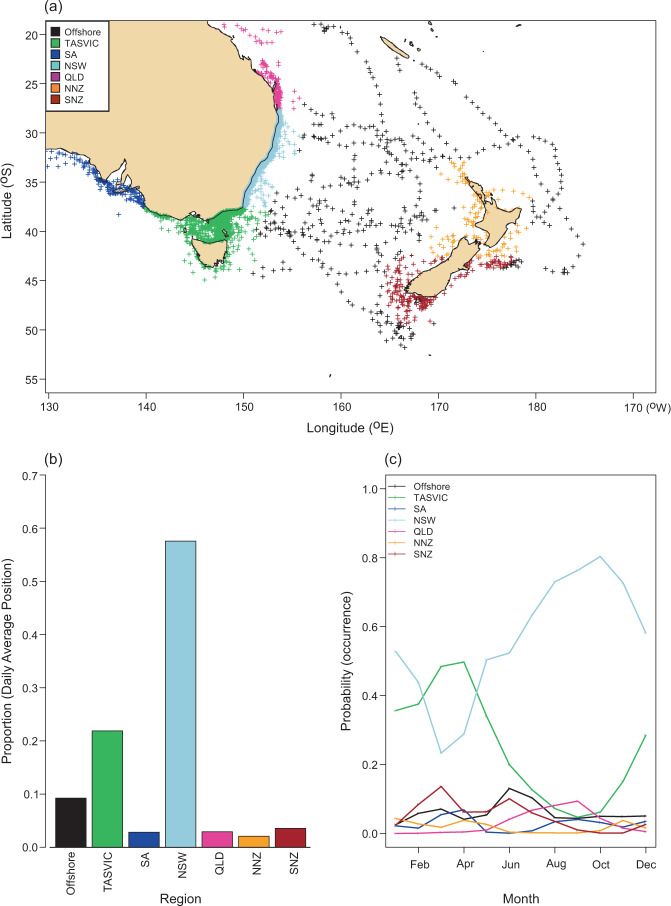
Regional and seasonal occurrence patterns. (**a**) Estimated Daily Average Positions (DAPs) for 87 satellite-linked radio and acoustic tagged white sharks off eastern Australia, coloured coded by region. (**b**) Proportion of time spent by region for eastern Australasian white sharks. (**c**) Seasonal probability of occurrence for eastern Australasian white sharks. Lines represent the estimated effect of month of year on the probability of a randomly selected individual occurring in any region. Regions: offshore = all areas >200 nautical miles from the coastline, TAS/VIC = Tasmania and Victoria, SA = South Australia, NSW = New South Wales, QLD = Queensland, N-NZ = North Island, New Zealand, S-NZ = South Island, New Zealand. Map was generated using the marmap package in R^67^.

Immature white sharks showed distinct resident and transiting regions. Of the seven defined geographical regions (see Methods) (Fig. 5a), New South Wales and Tasmania/Victoria, accounted for >80% of DAPs, with the New South Wales region alone accounting for >60% of all DAPs (Fig. 5b). Predictions of seasonal occurrence from a generalized additive model (GAM) (Fig. 5c) showed generally high probability of occurrence in New South Wales (probability of occurrence >0.5 in all month except February, March and April). The monthly progression of predicted seasonal occurrence indicated the highest probability of immature white sharks occurring in coastal waters of New South Wales during the austral spring (September-November). Peak occurrence of immature white sharks in coastal waters off Victoria and Tasmania, including within Bass Strait, was during the austral autumn (March – May) (Fig. 5c). While much lower than these two regions, probabilities of occurrence in Queensland peaked in August – September (presumably as part of residence in northern New South Wales waters); off the South Island, New Zealand in March and June; and in offshore waters in June. No obvious seasonal patterns were visible for coastal areas in South Australia or North Island, New Zealand.

### Intra- and inter-individual variability in movement patterns

A subset of the sharks tagged for this study exhibited movement patterns consistent with the three north-south migratory corridors previously proposed for the eastern Australasian population (Supplementary Figure S1), as well as several extensive east-west excursions into the habitat of the southern-western Australian white shark population.

**Figure 6:**
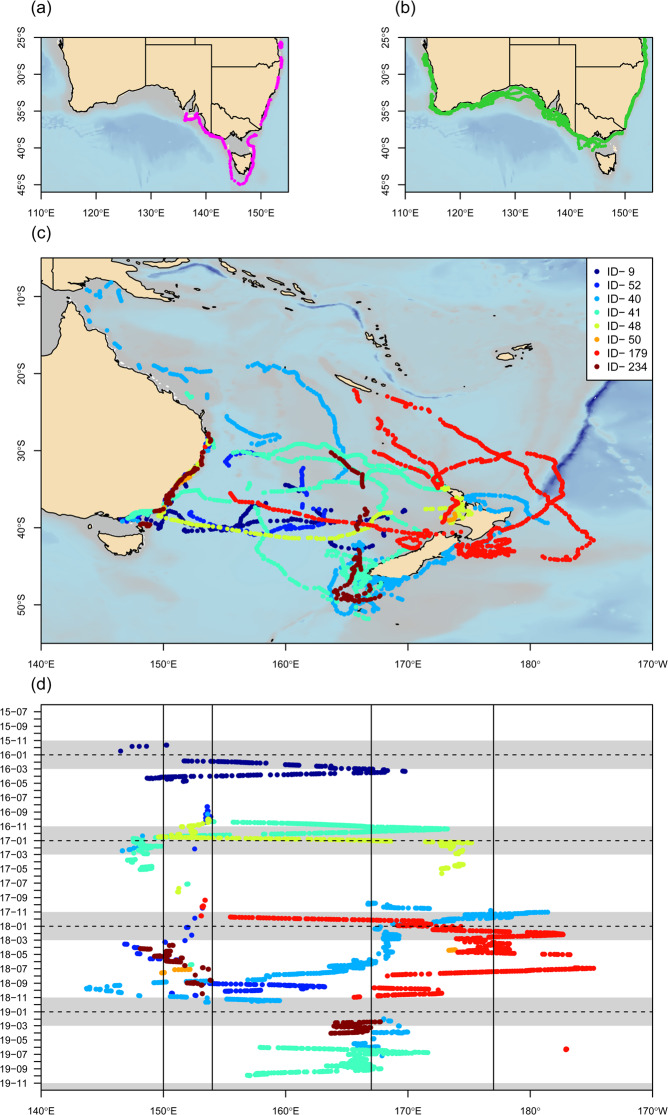
Broad scale movements and regional connectivity. (**a**) and (**b**) Estimated movements of shark 25 (pink line) and shark 28 (green line) showing movements west of Bass strait across the hypothesized line of separation between the eastern Australasian and southern-western Australasian sub-populations into the southern-western distributional range. (**c**) Large scale regional movements throughout the Tasman and Coral Seas from estimated tracks for all sharks (n = 8) that traversed to New Zealand. (**d**) Longitudinal positions by month for the 8 sharks that traversed east of the 160° longitude. Grey shading indicates the period between 01 November and 01 March; dotted horizontal line is at 01 January for each year; solid vertical lines bracket the coastal zones of Australia (150°E to 154°E) and New Zealand (167°E to 178°E). Maps were generated using the marmap package in R^67^.

Twenty-five sharks tagged in eastern Australia ventured into waters west of Wilsons Promontory, Victoria (39.0°S, 146.4°E) (Fig. 1). This region has previously been suggested as the geographical break which genetically divides the two Australasian white shark populations^26^. Twenty-three of these sharks extended their movements west to Cape Otway, Victoria (38.85°S, 143.53°E) (Fig. 2b), while two (nos. 25 and 28) ventured deep into the southern-western Australian distributional range (Fig. 6 a,b). Shark 28, which was tracked for a period of approximately 32 months, made at least two migrations between eastern and western Australia (Fig. 6b). The total estimated distance of these migrations exceeded 39,700 km. The most northerly positions where this individual was detected were Fraser Island, Queensland (25.25°S, 153.15°E) off the Australian east coast and Kalbarri, Western Australia (27.67°S, 114.15°E) off the west coast (Fig. 6b). Movements of shark no. 25 ranged from Coffin Bay in South Australia to Gold Coast, Queensland (28.02°S, 153.4°W) (Fig. 6a). Both sharks showed some degree of seasonal residence within the distributional ranges of both populations. Shark no. 28 remained in waters off Fraser Island, Queensland, for a period of 29 d and spent several periods, lasting up to four months, in the Great Australian Bight. Shark no. 25 resided at Kangaroo Island, South Australia for three months and along the northern New South Wales coast between Byron Bay (28.64°S, 153.61°E) and South West Rocks (30.88°S, 153.04°E) for periods of up to five months.

Eight (two female, six male) sharks traversed the Tasman Sea between Australia and New Zealand (Fig. 6c). In all cases where crossing occurred in the temperate waters of the Tasman Sea, the transits between Australia and New Zealand were generally directed and similarly rapid, with crossing times ranging from 23 to 38 d (Fig. 6d). Crossing speeds ranged from 2.4 to 3.8 km hour^−1^ (mean 3.0 km hour^−1^, SD 0.5). Two of the eight sharks traversing to New Zealand (shark nos. 52 and 234) subsequently returned to eastern Australia. Three others (nos. 41, 48 and 179) were tracked for extended periods of time ranging from approximately 21 to 35 months, covering a latitudinal range of 8 to approximately 52 degrees. The estimated total distance travelled for each of these sharks exceeded 5,700, 15,600, and 6,600 km for individuals 41, 48 and 179, respectively. Shark no. 41 migrated from New South Wales via New Zealand to Papua New Guinea across two consecutive years, arriving in the Gulf of Papua on 25 September in both 2018 and 2019 (data for 25 September 2019 was outside the general data collection period, hence only the 2018 track is presented in the figures). Sharks 48 and 179 each conducted several cyclic oceanic migrations between New South Wales and New Zealand (shark no. 48) and New Zealand and New Caledonia (shark no. 179).

## Discussion

This study corroborates observations of previous studies on white sharks from the eastern Australasian population^19,22^. Specifically, immature white sharks were primarily distributed in coastal waters for most of the time, exhibiting directed and rapid transits across the Tasman Sea between the east coast of Australia and New Zealand. The two previously identified juvenile nursery areas^19^ were confirmed by our results. The Port Stephens juvenile nursery appears to be even more extensive than previously reported, however, extending over a 160 km stretch of coastline between Forster (32.18°S, 152.51°W) and south of Terrigal (33.44°S, 151.44°W). Our data included larger (and presumably older) immature individuals than previous studies and may represent an ontogenetic range expansion. Moreover, seasonal occurrence patterns within both nursery areas showed longer occupation periods compared to those previously reported. Immature white sharks were predominantly present in the Port Stephens nursery area from December through to March, rather than September to January^19,22^. The high use-area in Victorian waters was mostly occupied from November – February, rather than January – May^19,22^.

The importance of the New South Wales coast for white shark populations is consistent with previous tagging studies^19,22^ and catch data^27^. Yet, statistical summaries of time spent by region from electronic tracking data are often affected by tagging location and season and track duration^28^. This can potentially result in spatial and temporal biases. Due to the limited occurrence of white sharks in other regions of the study area, 86 out of 87 individuals in this study, were tagged in New South Wales coastal waters. For short-duration tracks, this might have biased the estimates of probability of occurrence in the New South Wales region upwards. Moreover, deployment of tags was biased towards the months when white sharks were available for tagging, i.e. May – December (Supplementary Figure S2). Forty-four percent of all sharks were tagged in October, which might have imposed an upward bias on estimates of probability of occurrence during this month. When interpreting the results of our study, effects of tag deployment location, date and track duration must hence be taken into account. However, we argue that it is unlikely that temporal and spatial biases in this study are strongly influential: (1) While tracking durations were highly variable, ranging from 38 to 1816 d, nearly 65% of all sharks were tracked for durations >1 year. All of these sharks either returned to the NSW coast or never left this region during their tracking period. (2) Our analysis of seasonal migration cycles and residency patterns (Fig. 3) indicated peak abundances in NSW coastal areas from mid-October to late January. Although the majority of sharks  were tagged in October, negligible numbers were tagged in the months of November – January (seven sharks total, see Supplementary Figure S2). This indicates that tagging dates and the modelled probability of occurrence are either not correlated at all or so weakly that the correlation is not reflected in the model results.

The large number of  sharks tracked in this study improved our understanding of previously described occurrence and movement patterns and highlights the sensitivity of space use estimates to sample size^4^. Our findings emphasize that tagging studies of wide-ranging marine species must deploy adequate numbers of instruments within the time scale of interest (here within the annual cycle of seasonal migration) to capture key aspects of movement. While sharks tagged in smaller numbers (i.e. 25 prior to 2015) showed north-south migration patterns and were useful in establishing key aspects of the biology and spatial dynamics of juveniles in eastern Australia, clear overall seasonal patterns became much more evident only after the number of tagged sharks had doubled (Fig. 4). The increased sample size in this study also enabled us to define a more accurate picture of the spatial division between the eastern and southern-western Australian populations. Previous studies using smaller sample sizes demonstrated a clear delineation between the east−west movements of eastern Australasian white sharks through Bass Strait, with very few registered positions west of Wilsons Promontory, Victoria (Fig. 1)^19,20,22^. In our study, 25 sharks extended their movements farther west to Cape Otway, Victoria (38.85°S, 143.53°E) (Figs. 1, 2a). At least for immature white sharks there appears to be no fixed boundary, despite the genetic separation that has been observed in previous studies^26^.

The movements of two sharks confirmed connectivity between eastern Australasian and southern-western Australasian white sharks. Although, in a previous study, two juvenile white sharks had been tracked from South Australia to New Zealand and east Australia, respectively^20^, our study is the first to show east-west movements via Tasmania and Bass Strait into southern-western Australian waters. Using mitochondrial and nuclear markers, both individuals could be assigned to the southern-western Australasian population (Davenport *et al*., unpublished data). Genetic differentiation between the two populations has been demonstrated for maternally as well as biparentally inherited markers, indicating philopatry by males and females^26^. This finding is concordant with a previously observed lack of half-sibling-pairs between eastern Australasian and southern-western Australasian samples^29^. In our study, the documentation of only two sharks moving between eastern Australasian and southern-western Australasian populations, despite the large number of tags deployed, suggests that such movements are indeed relatively rare occurrences. Nonetheless, both sharks showed some degree of seasonal residence within the geographic extent of both populations, revealing connectivity between them. The discrepancy between dispersal and gene flow suggests that migrations between both populations either do not result in reproduction, or alternatively that the number of reproducing adult sharks that move between the two populations is lower than the genetic threshold above which the populations would be genetically similar^26^. Refinement of our understanding of mixing between eastern Australasian and southern-western Australasian populations will require additional research on the frequency of adult white sharks migrating between the two populations and the ecology and behaviour of adult southern-western and eastern Australasian white sharks in eastern and western Australian habitats, respectively.

The observed inter-individual variation in movement patterns is likely attributable to a combination of extrinsic and intrinsic factors. Of the 87 sharks tracked in this study, 22 dispersed occasionally into the pelagic environment for estimated periods of 5–75 d. Yet, only seven of these animals (8%) conducted directed long-distance migrations between eastern Australia, New Zealand, New Caledonia and Papua New Guinea. In contrast, migration frequencies of white sharks previously tagged in New Zealand were much higher, with 95% of tagged individuals migrating to south Pacific islands and/or the Australian east coast^21,23^. Although ~70% of sharks tagged in New Zealand were immature, those sharks were generally larger (FL range: 244–410 cm, mean 326 cm, SD: 53)^21,23,30^ than the sharks tracked in our study (FL range: 147–350 cm, mean 224 cm, SD 39), suggesting ontogenetic development of seasonal long-distance movements. Nonetheless, of the four sub-adults in our study, only one undertook a large-scale migration (shark no. 41 from New South Wales to New Zealand). The remaining three resided along the Australian east coast throughout the entire tracking period (746–1009 d). Such inter-individual variation within the same size-/age-classes has been reported by many studies tracking the horizontal movements of sharks^e.g.^^31–34^ and indicates that juveniles within the eastern Australasian population likely determine habitat use based on conditional strategies that are fixed upon a combination of intrinsic (e.g. age, physiological condition)^35,36^ and highly plastic extrinsic factors (e.g. variation in feeding niche, resource distribution, inter-annual oceanographic differences)^36–38^, rather than ontogeny alone. Oceanic travel speeds of the eight sharks crossing the Tasman Sea (mean 3.0 km^−1^, range 2.4 to 3.8 km hour^−1^) were remarkable similar to swimming speeds recorded in previous studies^20,22,23,39–41^ and within the range of speeds estimated to minimize the metabolic cost of transport^42^. While large, regional tracking datasets like ours can help to resolve what proportion of the study population conducts frequent long-distance migrations^43^, horizontal movement data alone cannot verify the proximate mechanisms of migratory behaviour. Future studies on juvenile populations should investigate potential underlying genetically determined differences in individual-level propensity for migration as well as the drivers of behavioural strategies that develop in early life history stages^31^.

Clear size and sex-based differences in movement patterns and habitat preferences of the sharks tagged in this study could not be identified. While generalized additive model (GAM) results suggested a size and sex-based relationship influencing probability of occurrence, further examination of the model predictions showed little evidence of difference in occurrence over the most numerous size ranges and lacked a clear signal of sex-based differences within these (see Supplementary Material for more details). We hence believe that the size range in our dataset is insufficient to put much weight on the model predictions. Nonetheless, it should be noted that eight of the ten sharks conducting long-distance movements were males, despite the five largest animals being females. Sex-based differences in migratory patterns of juvenile and adult white sharks have been observed throughout their range, e.g. the northeast and central Pacific^13,44^, Australia^45^ and South Africa^46^. Male-biased dispersal has previously been identified between Australasian and South African white sharks based on contrasting maternally and biparentally inherited genetic markers^47^. Our observations point to a male-biased propensity for large-scale migrations in immature white sharks, which is also supported by an inshore bias towards females, as observed in this (female/male ratio: 50:37) and previous work^22,48^. A female-biased preference for inshore habitats could result in juvenile females being more vulnerable to anthropogenic coastal threats, such as inshore fisheries and habitat degradation, than juvenile males. However, given the small number of long-distance migrants in this study, we must acknowledge the limitations of our sample size and the need to tag additional sharks to further test the hypothesis of juvenile male white sharks dispersing further than females.

Satellite data in this study is consistent with the hypothesis of several distinct migration corridors in the western South Pacific^20,21^ (Supplementary Figure S1). Paths taken by sharks nos.179 (Chatham Islands, New Zealand to New Caledonia) and 234 (southwest South Island, New Zealand, to southern Queensland) (Supplementary Figure S1) were remarkably similar to the south-north migratory routes observed in several sharks previously tagged in New Zealand waters^21,23,49^. Although departure and arrival dates were not synchronous among sharks utilizing the same migration routes, they did fall within the same seasonal periods^21,23,25,50^. This further highlights the existence of a strong temporal component to long-distance migrations^49^. Utilization of nearly identical travel paths by white sharks has also been reported in the north-east Pacific^51–53^ and by tiger sharks in Hawaii^54^ and along the Australian east coast^55^. Yet, the internal mechanisms underlying the ability of sharks to navigate between distant destinations remain to be unravelled^49,56^.

Overall, our tracking results have provided a more complete understanding of habitat utilization of immature white sharks across their eastern Australasian range and addressed a key uncertainty in the knowledge informing population management. By combining satellite and acoustic tagging data, we observed seasonal migration routes across multinational jurisdictional boundaries, highlighting the critical need for closely coordinated international management of this highly vagile species. The identification of high space-use areas and seasonal distributions lays the foundation for evaluating where, when and how these areas overlap with anthropogenic activities, including coastal development and commercial fisheries^14^, thereby fostering improved management practices. The long-term movement data obtained will also be useful for integration into spatially explicit stock assessment models^57^ that can provide regional estimates of management-related trends in white shark stock size and fishery impacts in the eastern Pacific. The large number of animals tracked in this study provides a sound basis for future studies aimed at disentangling the drivers behind the observed migration patterns, which will be critical for future conservation efforts. Data at this scale will also aid in predicting potential shifts in spatial distribution associated with global change.

## Methods

### Ethics statement

This work was carried out under permits issued to the New South Wales Department of Primary Industries (NSW DPI): Animal Care and Ethics Permits: ACEC 07/08 DPI and ACEC 14/07; Scientific Collection permit P07/0099; Scientific Research Permit P01/0059(A); Marine Parks Research Permit P16/0145-1.1 and Commonwealth Scientific and Industrial Research Organisation (CSIRO) Tasmanian Department of Primary Industries, Parks, Water and the Environment (DPIPWE): AEC Permit 22/2015-16, and its predecessors; DPIPWE Living Marine Resources Management Act 1995 Permit 15008 (and previous derivations), DPIPWE Threatened Fauna for Scientific Purposes permit 14239; Victorian Department of Environment and Primary Industries (VDPI) Research permit 10006912, VDPI Protected Aquatic Biota permit PA38. All tagging was carried out in accordance with the CSIRO Code of Practice for tagging marine animals^58^, with protocols used approved by the Tasmanian DPIPWE Animal Ethics and NSW DPI Animal Care and Ethics Research Authority.

### Tag deployment

Juvenile and sub-adult white sharks along the eastern Australian seaboard between Byron Bay and Corner Inlet, Victoria (38.77°S, 146.33°E) were dual-tagged with acoustic transmitters and satellite-linked radio transmitting (SLRT) tags. Acoustic transmitters (Vemco, V16-6H and V16-6X), with transmission intervals of 40–150 s and an approximate 6–10-year battery life were surgically implanted into the abdominal cavity following the general procedure of Bruce and Bradford (2013)^59^ and Heupel *et al*. (2006)^60^. SLRT tags, which transmit the shark’s position to the Argos satellite array whenever the dorsal fin breaks the surface, were mounted to the dorsal fin of each shark following the methods described in Bruce and Bradford (2012)^19^. In addition, each shark was tagged with a uniquely numbered identification tag (spaghetti tag, Hallprint, SA), which was inserted into the musculature at the base of the first dorsal fin for future visual identification. Three sharks were recaptured and fitted with an additional externally-attached acoustic transmitter as part of a wider research program. Sharks were either caught using Shark Management Alert in Real Time (SMART) drumlines^61^ or following protocols described in Bruce and Bradford (2013)^59^ and CSIRO (2015)^58^. Sharks were brought alongside the boat and secured with either an in-water sling or a belly and tail rope following capture. Prior to release, sharks were sexed and measured to the nearest cm for fork length (FL). Typically, sharks were restrained for less than 20 minutes before release. Each shark was assigned a unique ID number. Since all sharks in this study are part of a nationwide dataset, the numbering of individual sharks is inconsistent.

### Acoustic monitoring

Acoustic signals were tracked by 11 arrays of up to 36 passive acoustic receivers (Vemco VR2W and VR4G), in 10 different regions off the east/south-east and west coast of Australia and the east coast of Tasmania (see Fig. 1 for details on east/south-east coast receiver locations). Because receivers were operated by eight different organisations for a range of projects, the numbers, configurations and operational periods of each array varied over the course of the present study (Supplementary Table S2). Data were generally retrieved annually from VR2W receivers (biannually from Integrated Marine Observing System’s [IMOS] Animal Tracking receivers in some years) and weekly (until 12 September 2019) from satellite-linked VR4G receivers. All detection data reported below were collected from 08 October 2007 up to and including 12 September 2019.

### Track processing

Argos-derived tracking locations are subject to varying levels of spatial error, ranging from tens of metres to hundreds of kilometres^62^. To account for such measurement errors, as well as irregular sampling intervals, data were error corrected using a simple random walk state space model estimated using the Kalman filter/smoother, following the methodology detailed in Patterson *et al*. (2010)^63^. The Kalman filter used Argos-CLS positions, as per Patterson *et al*. (2010)^63^, but also assimilated acoustic detections and location information obtained during recapture events. Due to the high accuracy of acoustic positions relative to Argos-CLS positions, these were assigned an error at 10% of the error of the most accurate location class (Argos class 3). This effectively means that the Kalman filter treated the acoustic positions as having negligible error and constrained the estimated track to follow the acoustic detections exactly. The Kalman smoothing step interpolated the positions to regular 6 h time steps, which were used as the basis for the analyses detailed in the following sections. Note that acoustic data alone is not a viable input for the Kalman filter model which assumes error prone observations from a random walk as the data generating process^53^. Spatial locations from a finite set of fixed spatial locations does not accord with this model. Therefore, the acoustic detections were used as augmented high accuracy points within the time extent of a satellite track and were not used beyond 6 d of the last Argos filter location. Rates of movement during oceanic travel from Australia to New Zealand were estimated based on distances travelled from the Australian mainland to New Zealand and time spent travelling.

### Regional occurrence and models of seasonal use

For each individual shark we calculated the proportion of time spent within and extending ~ 200 nautical miles offshore of each Australian state and within 200 nautical miles of the New Zealand coastline. This method defined six ‘coastal’ regions; with a seventh region termed 'Offshore', for detections outside of the coastal regions. Following Harrison *et al*. (2018)^28^ we used a multinomial distributed generalized additive model (GAM^64^) fitted in the R library mgcv^65^ to quantify the presence of immature white sharks for the seven selected regions within the study area as a function of month of the year. The model can be written:

$${{\rm{M}}}_{0}:\,\Pr ({Y}_{i})=AL({\beta }_{0,s}+{{\rm{\beta }}}_{1,s}{\rm{Month}})$$

where *AL(h)* is the additive logistic transform which constrains the probability across categories *1, …, N* to sum to unity^66^.

To reduce the effect of missing data and to avoid relying solely on the Kalman filter interpolation, periods of more than 14 days between two Argos data points were removed from the data prior to inputting them into the GAM. Moreover, to reduce the number of data points included in the model, daily average positions (DAPs) were calculated from the 6 h Kalman filtered positions as described above. To determine a potential relationship between sex and size and the probability of occurrence in each region, we ran additional GAMs that included fork length and sex as variables (see Supplementary Material for details).

## Supplementary information

Supplementary Information.
