# Supplementary material for: Spatiotemporal distribution patterns of immature Australasian white sharks (Carcharodon carcharias)
Source: Sci Rep. 2020 Jun 23;10:10169. doi: 10.1038/s41598-020-66876-z (PMC7311443; doi:10.1038/s41598-020-66876-z)
Supplement: Supplementary file 1 — Supplementary Information. [file 41598_2020_66876_MOESM1_ESM.pdf]

Supplementary Information

Spatiotemporal distribution patterns of immature Australasian white sharks (*Carcharodon carcharias*)

Julia L.Y. Spaet[1, 2*], Toby A Patterson[3], Russell W. Bradford[3], Paul A. Butcher[2,4]

[1]Evolutionary Ecology Group, Department of Zoology, University of Cambridge, Downing Street, Cambridge CB2 3EJ, UK

[2] Southern Cross University, PO Box 4321, Coffs Harbour, New South Wales 2450, Australia

[3] CSIRO Oceans and Atmosphere, Hobart, TAS 7004, Australia

[4]Fisheries NSW, Department of Primary Industries, Sydney Institute of Marine Science, Mosman, New South Wales 2088, Australia

* Corresponding Author

Table S1. Details and tracking periods of 103 tagged white sharks. Detection period refers to the time period between date of first and last detection; total time at liberty refers to the time period between the date of tagging and date of last detection. FL: fork length; Ac: acoustic tag; NA: not applicable (shark was not fitted with tag type). ND: No Detections recorded. * indicates < 20 detections = shark was excluded from all analyses.

Shark ID	FL (cm)	Sex	Release location	Release date	First detection		Last detection		Detection Period		Time at liberty (d)
					SLRT	Acoustic	SLRT	Acoustic	SLRT	Acoustic	
1	187	F	Ballina, NSW	2015-08-26	2015-09-11	2015-09-07	2015-11-13	2015-11-16	63	70	82
2	187	F	Ballina, NSW	2015-08-26	2015-09-11	2015-12-05	2015-11-20	2017-10-16	70	681	782
3	276	F	Lennox Head, NSW	2015-08-26	2015-08-26	ND	2016-08-11	ND	351	ND	351
4	224	M	Ballina, NSW	2015-08-30	2015-08-31	2015-11-01	2016-08-26	2017-09-28	361	697	760
*5	270	F	Ballina, NSW	2015-09-08	2015-09-26	2015-10-26	2015-11-24	2016-01-18	59	84	132
6	226	M	Ballina, NSW	2015-08-28	2015-09-07	2015-09-26	2015-10-17	2015-10-06	40	10	50
7	270	F	Ballina, NSW	2015-09-12	2015-09-15	2015-10-21	2015-12-16	2016-06-24	92	247	286
8	200	F	Ballina, NSW	2015-09-12	2015-09-13	2015-10-07	2016-01-10	2015-11-16	119	40	120
9	272	F	Ballina, NSW	2015-09-13	2015-10-12	2015-11-17	2016-04-23	2015-12-16	194	29	223
*10	218	M	Ballina, NSW	2015-10-14	2015-10-17	2015-10-30	2016-02-18	2016-01-16	124	78	127
*11	260	F	Ballina, NSW	2015-10-15	2015-11-06	2016-02-14	2015-11-20	2017-05-24	14	465	587
*12	202	F	Ballina, NSW	2015-10-13	ND	ND	ND	ND	ND	ND	0
13	253	F	Lennox Head, NSW	2015-10-16	2015-10-17	2015-11-05	2016-08-02	2015-12-13	290	38	291
14	207	F	Ballina, NSW	2015-10-19	2015-10-19	2015-11-01	2016-02-11	2018-09-01	115	1035	1048
15	223	F	Evans Head, NSW	2016-05-31	2016-05-31	2016-06-12	2017-02-28	2017-09-20	273	465	477
16	245	M	Evans Head, NSW	2016-05-31	2016-06-01	2016-06-17	2017-09-23	2017-12-25	479	556	573
17	223	F	Evans Head, NSW	2016-05-31	2016-05-31	2016-06-04	2017-07-09	2016-09-29	404	117	404
18	255	F	Evans Head, NSW	2016-06-02	2016-06-03	2016-06-20	2016-08-15	2018-08-05	73	776	794
25	267	M	Ballina, NSW	2016-07-04	2016-07-06	2016-07-22	2018-09-03	2019-06-12	789	1055	1073
26	252	F	Ballina, NSW	2016-07-04	2016-07-04	2016-07-28	2016-10-21	2016-08-18	109	21	109
27	314	F	Ballina, NSW	2015-11-10	2016-07-04	ND	2017-11-25	ND	509	ND	746
28	267	M	Ballina, NSW	2016-07-05	2016-07-05	2016-08-27	2019-01-11	2018-01-24	1019	515	1019
30	192	F	Tuncurry, NSW	2016-07-21	2016-07-21	2016-07-27	2017-05-09	2019-03-15	292	961	967
31	235	M	Tuncurry, NSW	2016-07-21	2016-07-21	2016-08-02	2016-10-14	2019-04-28	85	999	1011
33	255	F	Tuncurry, NSW	2016-07-22	2016-07-22	2016-07-26	2017-01-03	2016-11-07	165	104	165
*34	201	F	Crowdy Head, NSW	2016-07-27	2016-08-13	ND	2016-09-11	ND	29	ND	46
35	259	M	Coffs Harbour, NSW	2016-08-01	2016-08-02	2016-09-14	2017-09-30	2018-10-14	424	760	804
36	190	F	Coffs Harbour, NSW	2016-08-01	2016-08-01	2016-08-03	2018-01-29	2019-03-26	546	965	967
37	234	M	Coffs Harbour, NSW	2016-08-02	2016-08-06	2016-08-03	2017-02-11	2016-11-24	189	113	193
38	193	F	Ballina, NSW	2016-08-09	2016-08-11	2016-08-09	2017-06-15	2018-09-21	443	773	773
39	277	F	Ballina, NSW	2016-08-09	2016-08-13	2016-08-09	2016-08-13	2017-01-27	1	171	171
40	230	F	Ballina, NSW	2016-08-09	2016-08-09	2016-08-09	2018-12-28	2018-12-17	871	860	871
41	300	F	Ballina, NSW	2016-08-10	2016-08-19	2016-09-10	2019-07-07	2018-12-18	1131	829	1140
42	174	F	Tuncurry, NSW	2016-09-06	2016-09-06	2016-10-25	2017-08-16	2019-08-17	344	1026	1075
44	192	M	Tuncurry, NSW	2016-09-07	2016-09-07	2016-09-18	2017-02-22	2018-10-21	168	763	774
45	258	M	Tuncurry, NSW	2016-09-07	2016-09-07	2016-10-05	2017-09-30	2017-10-11	399	371	399
47	147	M	Tuncurry , NSW	2016-09-08	2016-09-24	2016-10-01	2017-04-19	2016-10-31	207	30	223
48	252	M	Ballina, NSW	2016-09-27	2016-10-02	2016-10-01	2018-10-25	2018-11-22	1093	782	1098
49	160	M	Ballina, NSW	2016-09-28	2016-10-03	2016-11-28	2017-06-17	2019-06-27	257	941	1002
50	187	M	Ballina, NSW	2016-10-01	2016-10-01	2016-10-07	2017-05-21	2017-08-08	232	305	311
51	240	M	Ballina, NSW	2016-10-02	2016-10-02	2016-10-15	2017-09-29	2018-08-09	362	663	676
52	268	M	Ballina, NSW	2016-10-02	2016-10-16	2016-11-13	2018-08-03	2016-12-27	656	44	670
53	218	M	Ballina, NSW	2016-10-02	2016-10-03	2016-10-02	2017-08-26	2017-11-17	327	411	411
54	350	F	Ballina, NSW	2016-07-05	2016-09-29	NA	2018-07-05	NA	923	NA	1009
55	190	F	Ballina, NSW	2016-10-04	2016-10-04	NA	2017-03-03	NA	150	NA	150

56	257	M	Ballina, NSW	2016-10-06	2016-10-06	2016-11-18	2017-04-04	2017-11-08	180	355	398
57	222	F	Ballina, NSW	2016-10-08	2016-10-08	2016-11-06	2017-04-08	2019-08-30	182	1027	1056
58	192	F	Ballina, NSW	2016-10-08	2016-10-18	2016-10-09	2017-03-04	2018-02-06	137	485	486
59	187	F	Ballina, NSW	2016-10-04	2016-10-09	ND	2016-11-20	ND	42	ND	47
60	320	F	Ballina, NSW	2016-10-15	ND	2016-10-24	ND	2019-06-02	ND	951	960
96	245	M	Coffs Harbour, NSW	2017-05-30	2017-06-18	2017-06-10	2017-09-29	2019-08-17	120	798	809
103	220	F	Tuncurry, NSW	2017-06-06	2017-06-06	2017-06-10	2018-04-29	2019-08-11	327	792	796
*105	251	F	Tuncurry, NSW	2017-06-06	2017-09-02	2017-06-11	2017-11-27	2019-04-25	86	683	688
106	247	F	Tuncurry, NSW	2017-06-06	2017-08-14	2017-06-21	2018-02-26	2017-12-07	196	169	265
160	235	M	Forster, NSW	2017-08-17	2017-08-17	ND	2019-02-14	ND	641	ND	641
179	250	M	Angourie, NSW	2017-09-12	2017-11-17	2017-09-12	2019-06-18	2017-10-12	578	30	644
180	210	F	Yamba, NSW	2017-09-12	2017-09-12	2017-10-11	2018-01-08	2018-09-06	118	330	359
227	269	M	Forster, NSW	2017-10-24	2017-11-01	2017-11-12	2018-03-22	2019-05-11	141	545	564
229	224	M	Evans Head, NSW	2017-10-25	2017-11-01	2017-11-18	2018-09-05	2017-12-11	308	23	315
234	277	M	Ballina, NSW	2017-10-28	2017-11-20	2017-11-01	2019-02-28	2019-06-29	514	605	609
251	224	M	Coffs Harbour, NSW	2017-11-15	2017-11-15	2017-11-24	2018-01-10	2018-03-31	56	127	136
290	255	M	Crowdy Head, NSW	2018-07-27	2018-07-27	2018-07-29	2019-02-09	2018-09-28	428	61	428
315	176	M	Evans Head, NSW	2018-08-29	2018-08-29	2018-10-19	2019-02-20	2019-08-16	335	301	352
341	252	F	Crowdy Head, NSW	2018-12-04	2018-12-04	2018-12-12	2019-02-05	2019-07-27	187	227	235
*358	260	F	Lennox Head, NSW	2019-07-11	2018-04-19	ND		ND	486	ND	38
*359	186	F	Ballina, NSW	2019-07-11	2018-12-03	ND		ND	300	ND	80
*364	177	F	Tuncurry, NSW	2019-07-18	2019-07-18	2019-07-30	2019-08-13	2019-08-25	59	26	59
365	174	M	Tuncurry, NSW	2019-07-18	2019-07-30	2019-07-28	2019-08-29	2019-09-07	36	41	51
*366	193	M	Tuncurry, NSW	2019-07-18	2019-07-18	2019-08-08	2019-08-09	2019-08-27	74	19	74
*367	218	M	Tuncurry, NSW	2019-07-18	2019-07-19	2019-08-11	2019-08-20	2019-09-02	32	22	46
368	178	M	Tuncurry, NSW	2019-07-18	2019-07-18	2019-08-14	2019-08-20	2019-09-05	72	22	72
*375	227	F	Ballina, NSW	2019-07-27	2019-07-31	ND	2019-09-30	ND	65	ND	65
1000	260	F	Corner Inlet, Vic	2010-12-14	2010-12-25	ND	2012-06-01	ND	524	ND	535
1003	230	F	Hawks Nest, NSW	2008-10-28	2008-10-28	2009-05-25	2008-12-30	2011-04-26	63	701	910
1004	160	F	Hawks Nest, NSW	2008-10-28	2008-10-28	2010-01-04	2009-06-10	2011-03-05	225	425	858
*1006	200	F	Hawks Nest, NSW	2008-10-30	2008-10-30	2010-01-23	2008-10-30	2010-01-23	1	1	450
1010	190	M	Hawks Nest, NSW	2009-10-28	2009-10-28	2010-01-10	2011-09-08	2013-01-29	680	1115	1189
1011	220	F	Hawks Nest, NSW	2009-10-28	2009-11-01	2010-03-06	2010-03-16	2013-12-25	135	1390	1519
1012	210	M	Hawks Nest, NSW	2009-10-29	2009-10-29	2010-04-28	2010-01-17	2011-03-31	80	337	518
1013	190	F	Hawks Nest, NSW	2009-10-29	2009-10-29	2010-02-22	2010-01-03	2014-10-04	66	1685	1801
1014	220	F	Hawks Nest, NSW	2009-10-30	2009-11-01	2010-03-11	2010-04-22	2013-02-22	172	1079	1211
1015	200	F	Hawks Nest, NSW	2009-10-30	2009-11-07	2010-01-17	2010-01-26	2012-12-03	80	1051	1130
1023	220	F	Hawks Nest, NSW	2011-10-25	2011-10-29	2011-10-25	2011-12-28	2015-06-17	60	1331	1331
1027	210	M	Hawks Nest, NSW	2012-10-10	2012-10-12	ND	2013-03-16	ND	155	ND	157
1028	240	F	Hawks Nest, NSW	2012-12-19	2012-12-19	ND	2014-04-01	ND	468	ND	468
*1029	300	F	Hawks Nest, NSW	2012-12-20	ND	ND	ND	ND	ND	ND	ND
*1030	200	F	Hawks Nest, NSW	2012-12-19	ND	ND	ND	ND	ND	ND	ND
1041	220	F	Stockton, NSW	2007-10-10	2007-10-10	ND	2007-11-22	ND	43	ND	43
1042	230	F	Stockton, NSW	2007-10-11	2007-10-11	ND	2007-12-30	ND	80	ND	80
1043	240	M	Stockton, NSW	2007-10-10	2007-10-11	ND	2008-01-21	ND	102	ND	103
1044	180	F	Stockton, NSW	2007-10-11	2007-10-11	ND	2007-11-24	ND	44	ND	44
1045	170	M	Stockton, NSW	2007-10-11	2007-10-11	ND	2008-04-01	ND	173	ND	173
1046	170	F	Stockton, NSW	2007-10-08	2007-10-10	ND	2008-01-17	ND	99	ND	101
1047	190	M	Stockton, NSW	2007-10-08	2007-10-09	ND	2008-01-23	ND	106	ND	107

1048	180	F	Stockton, NSW	2007-10-11	2007-10-11	ND	2008-01-10	ND	91	ND	91
1050	220	F	Stockton, NSW	2007-10-11	2007-10-11	ND	2007-11-22	ND	42	ND	42
1051	170	M	Stockton Bight, NSW	2010-10-27	2010-10-27	2010-12-22	2011-02-05	2012-10-28	101	676	101
1052	200	M	Stockton Bight, NSW	2010-10-27	ND	2011-08-01	ND	2014-01-19	ND	902	1180
1054	190	F	Stockton Bight, NSW	2010-10-27	2010-10-27	2010-12-10	2011-01-02	2011-05-27	67	168	212
*1056	190	F	Stockton Bight, NSW	2010-10-27	ND	2010-12-07	ND	2010-12-07	ND	ND	41
1203	260	F	Hawks Nest, NSW	2012-10-10	2012-10-10	2013-02-03	2013-05-20	2017-09-30	222	1700	1816
1403	170	F	Hawks Nest, NSW	2014-11-24	2014-11-24	2014-12-01	2015-05-15	2018-06-30	172	1489	1496
1404	250	F	Hawks Nest, NSW	2014-11-24	2014-11-24	2014-11-29	2016-02-13	2017-10-21	446	1057	1062

Table S2. Characteristics of acoustic receiver arrays.

All receivers were manufactured by VEMCO. IMOS: Integrated Marine Observing System (www.imos.org.au).

Array	Configuration	Receiver	Number of receivers	Data collection period
Corner Inlet	Cluster	VR2W	4-10[A]	2011-10-13 - 2018-08-16
Estuarine Gates	Gates	VR2W	11-21[B]	2015-10-08 - 2018-09-09
Lobster Collectors	Cluster	VR2W	12-15	2016-09-07 - 2018-07-26
MARL	Cluster	VR2W	1-2 [A]	2016-10-20 - 2018-02-22
north-west Tasmania	Single unit	VR2W	1	2017-12-10 - 2018-01-16
NSW demersal offshore	Cluster	VR2W	1-19 [A]	2017-10-16 - 2019-02-14
Wolf Rock	Cluster	VR2W	1-30 [A]	2016-08-08 - 2019-01-17
Seacams	Cluster	VR2W	14-36[A]	2011-04-20 -2019-04-05
NSW offshore buoy	Along shore (offshore)	VR2W	1-30	2015-09-01- 2019-06-29
VR4G	Along shore (inshore)	VR4G	1-21[A]	2015-12-23 - 2019-09-08
IMOS receiver curtains	Curtain	VR2W	13-15 (CH)[C]	2010-02-10 - 2019-09-30
			15-30 (BL) [D]	2018-12-03 - 2019-09-30
			9-10 (N) [E]	2012-02-20 - 2019-09-30
South Australia	Cluster	VR2W	10-25[A]	2007-01-06 - 2019-09-30
Western Australia	Along shore (inshore)	VR4G	19-24 [A]	2009-01-01 - 2019-09-30

[A]The array configuration varied over the data collection period.

[B]The array configuration was modified by a gradual increase from 1 receiver in December 2015 to 21 receivers in July 2017.

[C]In 2015 the line was moved from North Solitary Island to South Solitary Island for logistical reasons. In 2016 the number of receivers was decreased from 15 -13; see Steckenreuter et al. (2016)[57].

[D]In 2009 the number of receivers was increased from 15-30. In 2017 it was decreased to 15 again; see Steckenreuter et al. (2016)[57].

[E]In 2009 the number of receivers was increased from 9-10. In 2014 it was decreased to 9 again; see Steckenreuter et al. (2016)[57].

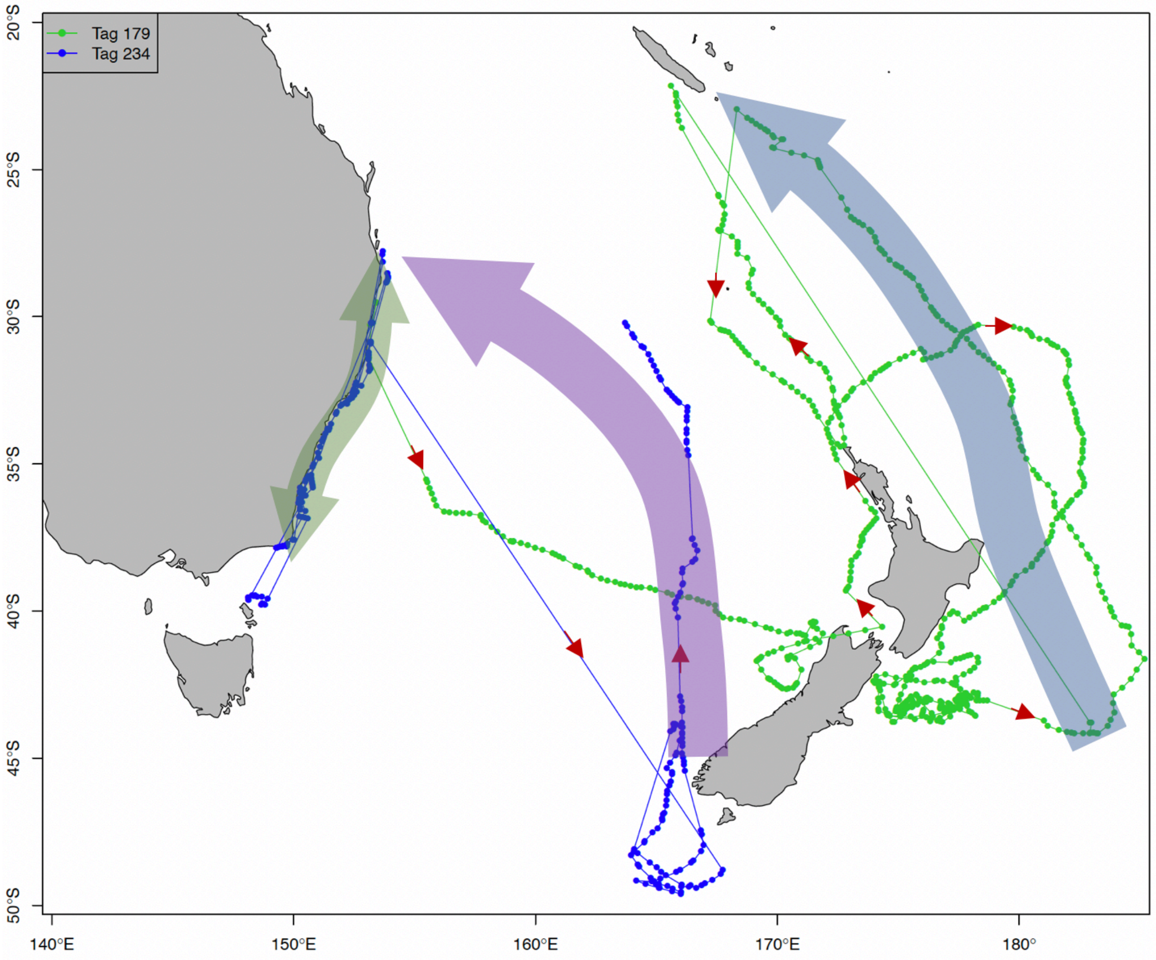

Figure S1. Estimated directional movements for shark 179 and shark 234, showing utilisation of three previously described migratory corridors. Green: Along the east coast of Australia; Purple: From southwest South Island New Zealand to southern Queensland; Blue: From the Chatham Islands, New Zealand along northeast North Island, New Zealand to New Caledonia. Red arrows indicate travel directions.

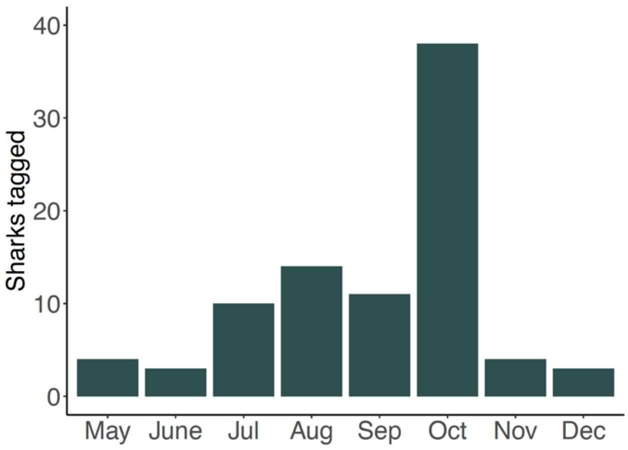

Figure S2. Cumulated number of individual white sharks tagged by month across the 2007-2019 study period.

Supplementary GAM information:

In addition to using generalized additive models (GAMs) to predict regional occurrence patterns (see methods section in the main document), we also used GAMs to determine the relationship between sex and size and the probability of occurrence in each of the seven defined regions. The response variable in these models was the 'region' (i.e. "NNZ", "NSW", "Offshore", "QLD", "SA", "SNZ", or "TASVIC") where a given Daily Average Position (DAP) was located (Table S3).

Table S3. Number of Daily Average Positions (DAPs) by month and region.

Region	Jan	Feb	March	April	May	June	July	Aug	Sep	Oct	Nov	Dec
NNZ	34	17	0	22	2	3	0	0	3	2	33	13
NSW	427	200	107	93	142	123	206	335	353	552	563	513
offshore	39	48	43	30	32	63	68	28	38	38	39	45
QLD	0	0	1	3	3	6	29	33	61	19	18	3
SA	18	7	22	29	0	0	2	16	19	22	14	31
SNZ	19	42	51	28	11	29	16	18	5	0	0	24
TASVIC	286	182	185	198	85	42	52	21	36	27	129	247

Several GAM structures were trialled on these data (see Table S4), which incorporated the following candidate predictors.

- Month – calendar month of the year
- FL – fork length (cm)
- FLcat – categorized fork length which was a factor variable indicating the size grouping. The categories spanned the data roughly equally with the following numbers of observations: N=1185 (130-189 cm FL), N=2834 (190 – 249 cm FL), N=2144 (250-299 cm FL).
- Sex – where the observations were split as female: N=3357, male: N=2986.

We tried several permutations of these variables in various models, the simplest containing only month. The results indicated that the highest deviance explained was from one of the most complicated models (see Table S4 mod5), which was also the most favoured based on AIC (Akaike's Information Criterion) value.

At face value, this would indicate that there is a size and sex-based relationship influencing probability of occurrence. However, examination of the model predictions showed little indication of difference in occurrence over the most numerous size ranges and little evidence of sex-based differences within these (Fig. S3).

While these results are noteworthy, we believe that there is an insufficient size range in the data to yield robust model predictions. The majority of the tagged sharks in this study were within the 190-250 FL cm size range (Fig. S4), representing approximately one single year class (given that white sharks grow ~ 50 cm a year as juveniles). Hence we conclude that the models are picking up a minute amount of size and sex differentiation in movement and perhaps that older/larger individuals are starting to move into a different migration regime. Crucially however, we do not believe that the satellite tagging data set analysed in this study is able to robustly predict what this altered movement regime actually is. We hence chose to only document the trends in occurrence probability through the year (see Fig. 5 main paper). While this is clearly a limited statistical model (see Table S4) and explains a low proportion of the deviance, it nonetheless captures the degree of residence for the juvenile age class represented in this data in each of the regions.

Table S4. GAM model structure and goodness-of-fit statistics

Code	Formula	Degrees of freedom	AIC	% Dev explained
mod0	~ s(month)	54.40	12826.83	10.9
mod1	~ s(month) + FLcat	62.95	12268.88	16.45
mod2	~ s(month) + Sex	57.73	12800.34	14.8
mod3	~ s(month) + FLcat + Sex	69.29	11802.96	19.4
mod4	~ s(month) + s(FL)	99.33	9976.69	32.9
mod5	~ s(month) + s(FL) + Sex	110.08	9413.36	36.5
mod6	~ s(month, by=Sex) + FLcat	104.80	11766.95	20.1

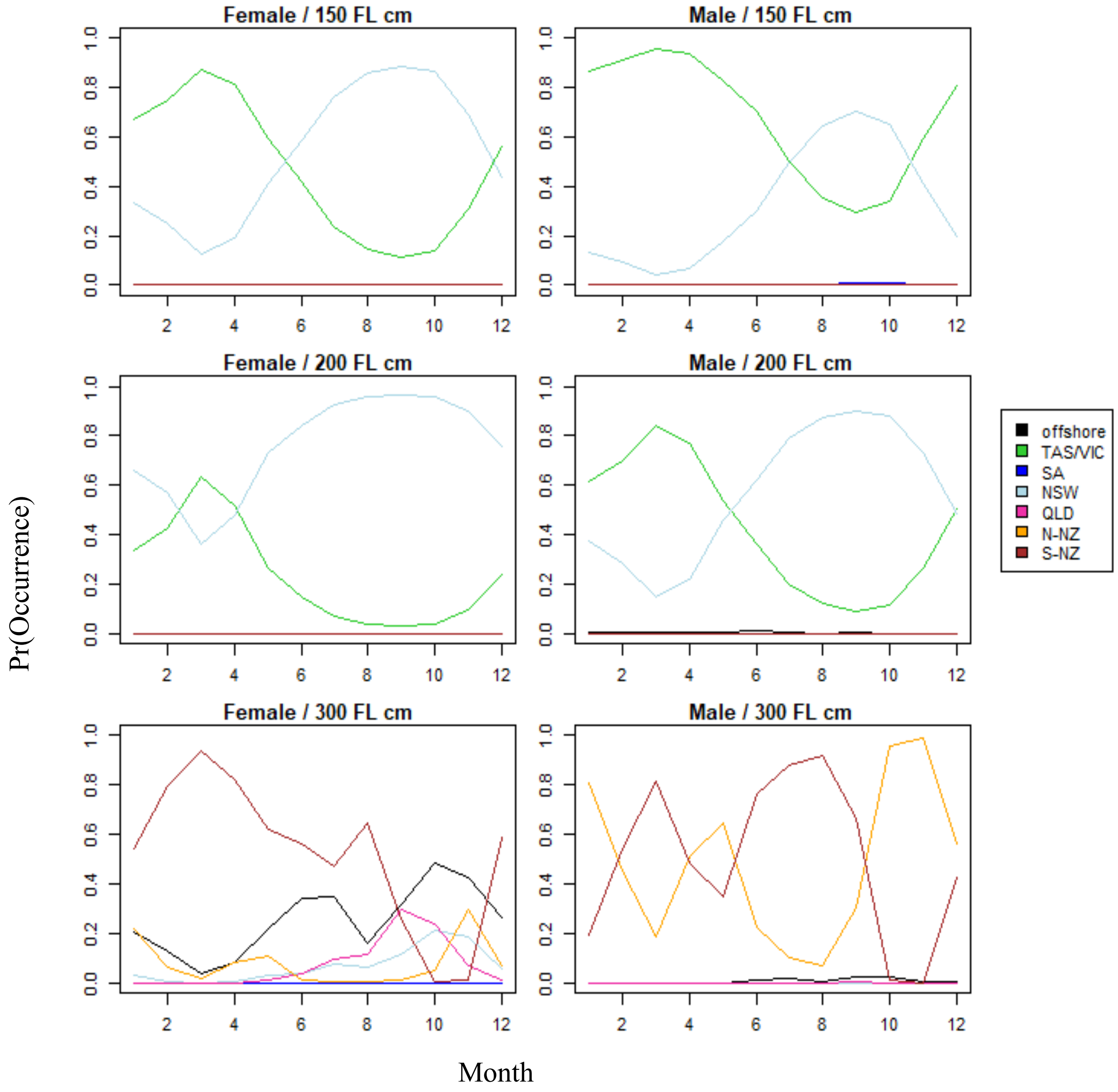

Figure S3. Probability of presence of immature Australasian white sharks in each of the seven defined regions (see Fig. 5 main text) by month of year, by sex and for four different example fork lengths (FL). The upper two rows (150- 200 cm FL) show a clear signal of presence alternating between NSW and TASVIC with a similar pattern throughout the year. The bottom row shows that offshore and New Zealand regions more distant from the NSW nursery areas become more prominent in larger (300 cm FL) animals and demonstrates a lack of the clear annual cycle observed in the smaller size classes.

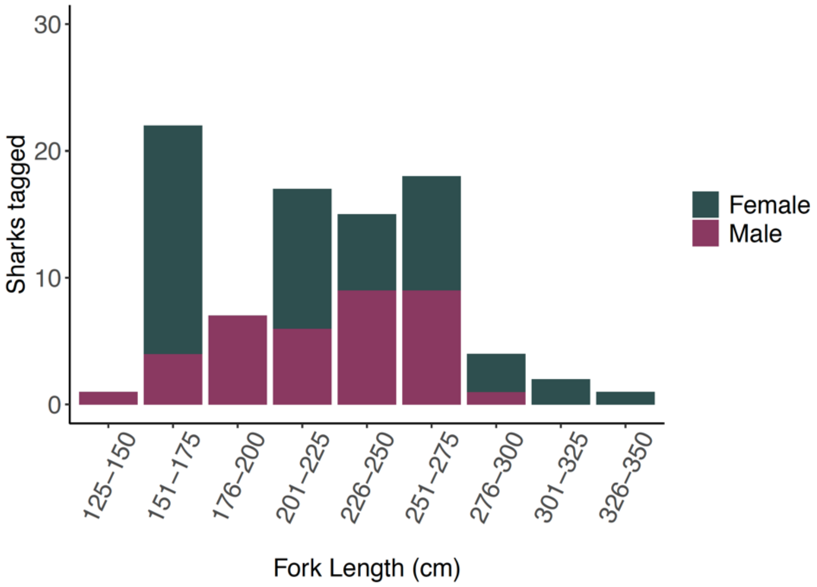

Figure S4. Size distribution of tagged sharks by sex.
